# Tailoring of corticosteroids in COPD management

**DOI:** 10.1007/s13665-014-0084-2

**Published:** 2014-07-06

**Authors:** Daan A. De Coster, Melvyn Jones

**Affiliations:** Department of Primary Care and Population Health, University College London, Upper 3rd Floor, UCL Medical School (Royal Free Campus), Rowland Hill Street, London, UK NW3 2PF

**Keywords:** Corticosteroids, COPD, Drug therapy, Fluticasone, Budesonide, Beclometasone, Ciclesonide, Mometasone, Salmeterol, Formoterol, Vilanterol, Tiotropium, Glycopyrronium, Inhaler, MDI, DPI, Phenotype, Heterogeneity, Withdrawal, Exacerbation

## Abstract

This literature review updates the reader on the new studies regarding steroid therapy over the last year in stable COPD and in exacerbations. In stable COPD, we critique the 2011 update and 2013 revision of the GOLD guidelines, discuss why combining inhaled corticosteroids (ICS) with long-acting beta-agonists (LABA) (ICS/LABA) is preferable over LABA alone and review the literature for intraclass differences, finding that the evidence does not clearly support superiority of any particular ICS/LABA. We also address other comparisons against ICS/LABA, including triple therapy. We briefly review which type of inhaler should be chosen. For exacerbations, we report the REDUCE trial findings favouring a 5-day course of systemic steroids, and other trials addressing which steroid and route to use, including in an intensive care setting. Lastly, the future lies in new anti-inflammatories and re-phenotyping the heterogeneous amalgamation of COPD. A Spanish guideline recommends distinguishing steroid-responsive eosinophilic exacerbators from other phenotypes.

## Introduction

Chronic obstructive pulmonary disease (COPD) is a disease characterised by chronic airflow limitation and airways inflammation. It is a leading cause of morbidity and mortality worldwide, and this is set only to increase. A patient may present with dyspnoea, chronic cough and sputum production, and a history of exposure to risk factors such as smoking. The diagnosis, however, is spirometric, with a post-bronchodilation forced expiratory volume in one second (FEV1) to forced vital capacity (FVC) ratio < 0.70. The aim of COPD management is to slow down the progression of the disease and improve the patient’s quality of life [1•].

Corticosteroids are thought to decrease the chronic inflammation in the bronchial tree, thereby improving obstructed airflow.

Inhaled corticosteroids (ICS) in COPD are known to improve symptoms, lung function, quality of life, and reduce exacerbations. They do not, however, modify the long-term decline in lung function nor mortality. Associated adverse effects are pneumonia, oral candidiasis, hoarse voice, bruising, and some mixed evidence regarding osteoporosis [1•]. There may be an increased risk of TB and non-tuberculous mycobacterial infections (Sabroe 2013), but ICS may reduce the risk of lung cancer [2–4].

Combining ICS treatment with long-acting beta2-agonists (LABA) seems to reduce mortality, despite increasing risk of pneumonias, but have no other significant adverse effects. Compared to the individual components, ICS/LABA improves lung function and reduces exacerbations even more in moderate to very severe COPD. [1•] Therefore, ICS are now only recommended as combined treatment, mostly with LABA, based on low spirometry values (American College of Physicians, ACP), symptom severity and risk of exacerbations (UK National Institute for Health and Care Excellence, NICE), or both (Global Initiative for Chronic Obstructive Lung Disease, GOLD) [5]. There have been some developments during the last few years in the evidence for triple therapy (adding in an additional drug, tiotropium, a long-acting anticholinergic/antimuscarinic [LAMA]).

Long-term treatment with systemic steroids, however, is associated with many adverse effects, especially steroid myopathy [1•]. They are recommended as a short course in the treatment of exacerbations of COPD because they shorten recovery time and the length of hospital stay, improve FEV1 and hypoxemia (PaO2), and reduce the risk of early relapse and treatment failure. In this area too, there have been some developments during the last few years.

## Aim & Structure

The aim of this review is to update the reader on the management of COPD with steroids with new developments from 2013 onwards.

We will update the reader on the GOLD 2013 revision and updated Cochrane reviews. We will review the indications and risks of inhaled steroids, which steroid or combination is best to use, and which inhalers (pressurised metered dose [pMDI], dry-powder [DPI]) to choose. For oral steroids, we will recount important new evidence on the duration of exacerbation treatment. Lastly, we will update the reader about new evidence regarding corticosteroid treatment responsiveness.

## Methods

We performed a Pubmed (MEDLINE) search with the search terms:

- ("**steroids**" [**MeSH Terms**] **AND** "**pulmonary disease**, **chronic obstructive**" [**MeSH Terms**]) **OR** (**corticosteroid copd**) **OR** (**steroids copd**) Filters: **Publication date from 2012**/**10**/**01**
- (Current as of 31/03/2014)

For head-to-head comparisons of inhaled steroids, we performed searches of both Pubmed and ClinicalTrials.gov for recent trials, using two of the following: “budesonide”, “fluticasone”, “beclomet(h)asone”, “ciclesonide”, “mometasone”, “flusinolide”, “betametasone”, or “triamcinolone”, combined with “COPD”. (These latter searches are current as of 17 January 2014.)

## Stable COPD: inhaled corticosteroids

### GOLD guidelines

#### 2011 Update

GOLD attempt to provide international guidelines for the management of COPD. A major update was produced in 2011, where it was felt that basing management decisions on spirometric staging alone (stage 1, mild as FEV1 ≥ 80 % predicted; stage 2, moderate FEV1 < 80 % predicted; stage 3, severe FEV1 < 50 % predicted; and stage 4, very severe FEV1 < 30 % predicted) showed insufficient prediction of disease severity. Instead, symptom scores, using the COPD Assessment Test (CAT) and modified British Medical Research Council questionnaire (mMRC), and exacerbation risk prediction (using the spirometry staging or the past year’s number of exacerbations) were recommended to divide patients in four GOLD groups. Groups A and B correspond to low exacerbation risk, with few and more symptoms, respectively. Groups C and D correspond to high risk (stages 3/4, and/or ≥ 2 exacerbations annually), with GOLD group C denoting few symptoms (CAT < 10 or mMRC grade 0 to 1), and group D more symptoms (CAT ≥ 10 or mMRC ≥ 2) [1•].

#### Criticisms

During the last 3 years, the GOLD guidelines have been criticised somewhat. Lee et al. [5] note that GOLD is funded by pharmaceutical companies that make COPD medications and that the board of directors, committee members, and reviewers have ties to the industry. Further, they note that there is no evidence that these guidelines produce better outcomes than the aforementioned ACP or NICE guidelines. Yawn [6] critiques that “the recommended medication hierarchy relies on research evidence from very short-term, 12-week studies and significant expert opinion.” She further wondered why validated primary care tools were left out in favour of the mMRC and CAT. Jones et. al [7] considered the new classification too complicated for primary care and were disappointed at the lack of consultation with primary care members. Perhaps the biggest criticism came from the COPDGene prospective cohort study: across a population, there are relatively few patients who correspond in the new group C, and also differences in classification, depending on whether the St George’s Respiratory Questionnaire (SGRQ) (as a surrogate for CAT) or the mMRC scale is used [8]. A Spanish cohort examining four scales (CAT, mMRC, BODE, CCQ) reached the same conclusion [9].

The GOLD authors responded that the CAT and mMRC were evidence-based, quick, and easy-to-use. They conceded, however, that “…in 2011 there were no data concerning [CAT scale] equivalence with mMRC. These data are now available and show the equivalence threshold for CAT ≥10 is mMRC ≥1 [not ≥2], something that might need to be addressed in future GOLD updates." [10]

#### 2013 Revision

February 2013 saw the “first update of the 2011 revised report" [1•]. The CCQ (Clinical COPD Questionnaire) was added to the CAT and mMRC, with a cut-off of 0-1 for groups A and C, and > 1 for groups B and D.

There were some minor changes to the stable COPD treatment hierarchy, and the most important change was that the first line for group D now includes the option of triple therapy, due to new evidence (see further below). Changes are outlined in Table 1.

**Table 1:** Revised GOLD 2013 management of stable COPD. **Differences with 2011 update in bold**

GOLD group	First choice	Alternative choice (in random order)
A (low risk, less symptoms)	SABA or SAMA	LABA or LAMA or SABA + SAMA
B (low risk, more symptoms)	LABA or LAMA	LABA + LAMA
C (high risk, less symptoms)	ICS/LABA or LAMA	LABA + LAMA **or LABA** + **PDE**-**4I or LAMA** + **PDE**-**4I**
D (high risk, more symptoms)	ICS with (LABA **and**/or LAMA)	ICS/LABA + LAMA or ICS/LABA + PDE-4I or LABA + LAMA or LAMA + PDE-4I **or ICS** + **LAMA**

SABA, short-acting beta-2 agonist (e.g. salbutamol, terbutaline). LABA, long-acting beta-2 agonists (formoterol, salmeterol; extra-long: indacaterol, vilanterol). SAMA, short-acting anticholinergic (ipratropium, glycopyrronium, aclidium). LAMA, long-acting anticholinergic (tiotropium, umeclidinium). ICS, inhaled corticosteroid (e.g. budesonide, fluticasone, beclometasone, mometasone). PDE-4I, phosphodiesterase-4 inhibitor (e.g. roflumilast)

Reviewing the groups to which steroids are relevant – GOLD recommends as first choice for group C a fixed combination of ICS/LABA or a LAMA, with differentiation between these two choices difficult. Alternatives recommended are a LABA + LAMA or ICS + LAMA (although there is no evidence for this combination), as both LABAs and LAMAs reduce exacerbations. Further alternatives are suggested in cases of chronic bronchitis or low-resource settings.

For group D, ICS + LABA or ICS + LAMA is recommended, with some evidence for triple therapy (ICS + LABA + LAMA). It is the first thing to try as an alternative, with phosphodiesterase-4 inhibitors (PDE-4I) to be added in cases of chronic bronchitis, and more alternatives suggested if LABAs are unacceptable choices.

### ICS/LABA versus LABA

Inclusion criteria of randomised controlled trials (RCTs) are often based on the guidelines preceding their protocol. The difficulty with changing guidelines significantly is that one has to review the old evidence for the applicability of their patient populations.

#### Group C or D patients: ICS/LABA versus LABA

When group C or D patients present to clinicians, is GOLD justified in recommending an ICS/LABA as opposed to a LABA (indicated for group B)?

An updated version of the ICS/LABA versus LABA Cochrane review was published in 2012, with a search current as of November 2011 [11•]. The vast majority of the patients in the 15 included trials (n = 11,794 patients) had severe to very severe COPD (group C or D). Reviewing the inclusion criteria, at least the three biggest trials (48 % of n) had an mMRC of 2 (or equivalent). Like the GOLD guidelines, therefore, the evidence comes from group C, with preponderance for group D. Four new studies were included since the last version – four out of the top seven biggest in the review (37 % of n) – but this had little impact on the conclusions. Compared with LABA, the combination treatment was more effective in improving health-related quality of life, dyspnoea, symptoms, rescue medication use, and FEV1. There was low quality evidence that the combination treatment reduced exacerbation rates (rate ratio 0.76; 95 % CI 0.68 to 0.84), downgraded because of inter-study heterogeneity and by high withdrawal rates. In response to some criticism regarding the reporting of exacerbations in trials with high drop-out rates [12], a new odds ratio was reported for patients with at least one exacerbation, also showing improvement in favour of ICS/LABA (OR 0.83; 95 % CI 0.70 to 0.98). There was no difference in mortality or hospitalisations. Importantly, however, there was an increased risk of pneumonia (OR 1.55; 95 % CI 1.20 to 2.01), which was not significantly different in trials with different doses. This was deemed moderate quality evidence, again due to the high withdrawal rate.

The evidence that ICS decrease the risk of exacerbations, yet increase the chance of pneumonia is corroborated by the 2012 update of the ICS (alone) vs. placebo Cochrane review [13]. As it also, however, found that there was no difference in mortality, so there have been suggestions in the past that ICS might increase all pneumonia events, but reduce severe pneumonias and their complications, as Almirall et al. [14] explain. Their 1-year observational study found that ICS had no impact on hospitalisation for community-acquired pneumonia when adjusting for severity, and had no association with ICU admission, days-to-clinical recovery or mortality. This is disputed by another publication from last year, a claims-data analysis (data from 2006-2010). It found that compared to no treatment with ICS, ICS use was associated with a significant, potentially dose-related increase in risk of pneumonia in patients with COPD. The authors defended the methodology of using a non-RCT design, stating that this association was unlikely to be down to more frequent use of ICS in patients with exacerbations, as exacerbations are usually viral and that the article’s real-life population had more comorbidities, reflecting real-life, and higher incidence of pneumonia: "This suggests the possibility that the incidence of ICS-associated pneumonia among non-selected patients with COPD may be higher than reported in controlled clinical trials." [15]

Another recent cohort study, however, with data from 1990 – 2005, followed up patients until 2007 or their first serious pneumonia event. They did find that ICS were associated with an increased risk of serious pneumonia by 69 %. The higher risk was prolonged with long-term use and disappeared about 6 months after discontinuation. This risk was particularly raised for fluticasone (RR 2.01) (also dose-related), much higher than for budesonide (RR 1.17) [16].

This raises the question whether there are intraclass differences in the effects of steroids. A 2009 individual patient data meta-analysis examining budesonide did not find an increase in pneumonia risk for this steroid [17]. Returning to our ICS/LABA vs. LABA question, the Nannini Cochrane review [11•] included only studies with the fluticasone propionate plus salmeterol (FP/S) and the more recent budesonide plus formoterol (BUD/F) combination inhalers. They performed a sub-analysis for these two separate products and found that there were no significant differences in either exacerbations or pneumonia. They did, however, find that candidiasis (OR 3.75) and upper respiratory infection (OR 1.32) were more frequent in FP/S than salmeterol only. For BUD/F, candidiasis adverse event data was not combined as the results were very inconsistent.

A new Cochrane review has sought to address whether the risk of pneumonia is increased in COPD when treated with steroids (alone or in combination with LABA) [18]. It includes 43 studies, 26 of fluticasone (n = 21,247) and 17 involving budesonide (n = 10,150). Fluticasone treatment had a higher risk of serious pneumonias requiring hospital admission (OR 1.78, 95 % CI 1.50-2.12; high quality), and this finding was not dose-related, or reduced when combined with salmeterol or vilanterol. Budesonide also had this effect, but to a lesser extent (OR 1.62, 95 % CI 1.00 to 2.62; moderate quality), and this effect was dose-related. The only difference the authors found when comparing monotherapy of fluticasone with budesonide was an increased risk of any pneumonia (including mild community cases) (OR 1.86, 95 % CI 1.04 – 3.34), but this is an indirect comparison, and there were inconsistent diagnostic criteria for pneumonia in the studies.

The third ICS/LABA combination product that is vying for market approval in COPD is the beclometasone dipropionate/formoterol (BDP/F) inhaler. A new Cochrane review was published looking into the effect of BDP in COPD [19], and includes the BDP/LABA vs. LABA comparison. In this review arm, only one study was found (n = 476, group C/D patients) [20]. The review found improvements in lung function and use of rescue medication, which were probably not clinically significant. No difference was found in pneumonia, mortality, or exacerbations. There was some debate concerning a significant increase in exacerbations leading to hospitalisation, depending on whether you accept the rationale of the author’s post-hoc analysis. Interpretation of these findings based on one study is difficult, and the review identified an ongoing trial looking at BDP/F vs. F which could shed more light on the matter (FORWARD study [21]).

Lastly, a new Cochrane network meta-analysis demonstrated that within the available evidence, ICS/LABA shows the biggest improvements compared with placebo, followed by LAMA or LABA, and then ICS alone, for both the SGRQ and FEV1 values [22]. They did not analyse other clinically important outcomes such as mortality and exacerbations, quoting two other meta-analyses on these instead: Dong et al. [23] found that ICS/LABA had the lowest risk of mortality when compared with placebo, tiotropium or LABA only, and a 2009 meta-analysis looking at exacerbations only, which found that neither ICS or ICS/LABA was more effective than each other or LAMA [24].

### Which inhaled corticosteroid?

The suggestion of differences between the available steroids and steroid combination products begs the question of which drug to prescribe as clinicians. Let us examine the available evidence.

#### Fluticasone propionate/salmeterol versus budesonide/formoterol

The best comparison of fluticasone propionate/salmeterol and budesonide/formoterol is a 2011 indirect, systematic review of RCTs of either FP/S or BUD/F, synthesised using the placebo comparator as a common denominator, as “there were no head-to-head trials at this time” [25]. Its results support the hypothesis that BUD/F is associated with fewer pneumonia events than FP/S. Apart from the indirect methodology, the authors advise caution due to heterogeneity, dominance by a single study (TORCH), and a lack of predefined diagnostic criteria for pneumonia.

There have been some small (≤4 weeks) interventional trials that compared FP/S and BUD/F. These studies are less clinically meaningful, as they cannot comment on effects on exacerbations. One study was a 1-week, double-blinded, cross-over trial (n = 442) of FP/S (50/500 μg twice daily [BD]) vs. BUD/F (320/9 μg BD), both by dry-powder inhalers (DPI). Both short-term treatments were effective, but BUD/F had a more rapid onset of effect compared with FP/S and “resulted in greater improvements in ability to perform morning activities despite the lower inhaled corticosteroid dose” [26]. A cross-over study of 22 patients investigated adrenal effect only. Comparing two 4-week treatment arms, no significant difference was found [27]. There was no wash-out period before the cross-over.

This year, new observational data were published too. A 10-year matched retrospective cohort, the PATHOS study, found that fluticasone/salmeterol was worse than budesonide/formoterol for the risk of pneumonia and pneumonia related events in COPD. [28] Jabbar [29] comments on the study, noting that the event rate “was higher in the fluticasone/salmeterol group to start with and this was not adjusted for in the analysis. Differences may be related to differences in the immunosuppressant potency between budesonide and fluticasone”. There have been a number of previous cohort studies. One was a 1-year cohort [30], which found that BUD/F was significantly less likely to have an emergency department visit or hospitalisation. Another study was a retrospective cohort study of US patients. Of 6,770 patients, equally divided, significantly fewer BUD/F patients had claims for SABAs, but not for LAMAs, COPD-related outpatient visits, or exacerbations, so that during 6 months after starting ICS/LABA therapy, there were no differences in their COPD medical costs [31]. We echo the conclusions of Blais et al. [30] and Jabbar [29] that long-term clinical trials are needed to draw certain conclusions and rule out confounders.

#### Budesonide/formoterol versus beclometasone/formoterol

There is relatively few data comparing the budesonide/formoterol combination (DPI) and beclometasone/formoterol inhalers (which are usually pMDI), probably as both combinations are quite recent.

The previously mentioned Calverley RCT [20] also contained a BUD/F arm; therefore, it allows us to compare BUD/F 400/12 μg DPI with BDP/F 200/12 μg pMDI (n = 479). During 48 weeks it showed no significant differences, with both improving lung function, exacerbation rates, quality of life, and symptoms, with no safety concerns.

Most recently, a small blinded cross-over RCT of 28 COPD patients found that after single administration, there was no difference in the onset of bronchodilation between BDP/F 200/12 μg Modulite (pMDI) and BUD/F 320/9 μg Turbuhaler (a DPI) [32].

#### Fluticasone propionate/salmeterol versus beclometasone/formoterol

Comparing fluticasone propionate/salmeterol with beclometasone/formoterol, there has been one small RCT (n = 18) comparing 12 weeks of FP/S 500/100 μg/day or BDP/F 400/24 μg/day. BDP/F actually showed a significant reduction in air trapping and a clinically significant improvement in transition dyspnoea index, which was not seen for FP/S. BDP/F also showed a significantly greater reduction in residual lung volume than FP/S [33]. Again, these outcomes are clinically not very relevant, but hint at how the drugs are working. This trial was also not powered for adverse events. However, an observational cross-sectional study in asthma and COPD found that beclometasone use (not necessarily in a combination inhaler) was significantly negatively associated with oropharyngeal side-effects, whereas fluticasone was significantly positively associated [34]. We found a completed, but not yet published, 12-week RCT comparing the same dosages as Tzani et al. [33] which will hopefully give more clarity [35].

#### Fluticasone furoate/vilanterol

Fluticasone furoate (FF) may have a better lung absorption than fluticasone propionate [36]. A new combination product, fluticasone furoate/vilanterol (FF/VI), may improve adherence in some patients, as it is the first once-daily (OD) ICS/LABA and is delivered through a new DPI device (Ellipta), which is said to be easier to use [37]. Just like the BPD/F inhaler, FF/VI has not been included yet in the Nannini review [11•]. Examining its effectiveness over vilanterol alone, therefore, four trials have been published. Two identical 1-year RCTs (n = around 1,630 each) in moderate to severe COPD of exacerbators, compared VI 25 μg with VI 25 μg combined with FF 200 μg, 100 μg and 50 μg. Their pooled analysis showed a significant reduction in exacerbations for all the combination groups compared to vilanterol alone. However, pneumonia and fractures occurred more commonly, and eight pneumonia deaths occurred in the FF/VI groups compared to none in the VI-only group [38]. The publication was critiqued by Morjaria and Morice [39] for not including comparison with the standard twice-daily dosing FP/S, the pre-study ICS use in most participants, including in the VI-only study arm, and the high findings of pneumonia. The authors responded that, although difficult, any comparisons with FP/S indicate possible reductions in exacerbation rates, that ICS use did not affect the outcomes as there was no difference in withdrawal rate between groups during the first month and a persistent effect on exacerbations after that, and that the pneumonia rates were similar to those described with other ICS and still ten times lower than the reported exacerbation rate [40].

A 24-week RCT of FF/VI (100/25 μg, 50/25 μg) vs. individual components (FF 100 μg, VI 25 μg) and placebo was published last year [41]. All active treatments improved lung function, but there was no significant difference between FF/VI dosages and VI alone. A trial of the same design, but with double the FF/VI doses, found the same results [42]. Both trials had no safety concerns with FF/VI over VI. The year before, two studies had demonstrated FF/VI’s superiority to placebo [43, 44]. There have also been two 1-week long safety trials to examine the safety of FF/VI in hepatic and renal impairment respectively; [45] only in hepatic impairment is caution advised, as there is a higher risk of systemic corticosteroid side-effects.

#### Fluticasone propionate/salmeterol versus fluticasone furoate/vilanterol

The first trial comparing fluticasone furoate/vilanterol with fluticasone propionate/salmeterol, used FF/VI 100/25 μg OD or FP/S 500/50 μg BD over 12 weeks. Improvements in lung function and health status were not significantly different and there was no significant difference in safety profiles [46]. There is a running 12-week study that is evaluating the effects of FF/VI 100/25 μg against a lower dose FP/S combination (250/50 μg BD) [47]. A similar trial in asthma has demonstrated non-inferiority in lung function and safety profile [48].

#### Ciclesonide and mometasone

Ciclesonide and mometasone are two other ICS that have the potential for once-daily administration in COPD, due to their long-acting nature [49, 50]. In both COPD and asthma, however, these drugs are infrequently prescribed in comparison to the more familiar ICS [51]. Concerns were raised last year that ciclesonide seems to be more lipophilic than budesonide in mice models, which theoretically could increase systemic adverse events [52]. For mometasone, however, there is a lot of safety data from asthma [53], justifying its use in a new combination product.

#### Mometasone/formoterol

The most recent ICS/LABA combination is mometasone furoate/formoterol (MF/F), also not yet included in the Nannini review [11•]. Two 26-week trials of identical design (total n = 2,251) were combined in one analysis before unblinding of either’s results. It compared MF/F 400/10 μg, MF/F 200/10 μg, MF 400 μg, F 10 μg, and placebo. There was significant superiority to placebo on most outcomes, but it is more useful to again examine the Nannini review question. Both MF/F doses showed significantly greater increases in spirometry than F 10 μg alone throughout the trial. The effect was four-fold greater with MF/F 400/10 μg than with F 10 μg. No difference was found in improvements of symptoms. There was a 26-week follow-up extension for 75 % of patients. The incidence of exacerbations was lower in the MF/F groups, including for severe exacerbations. All arms had few pneumonia incidences (≤2 %). The rate of adverse events did not differ significantly over the 26 or 52 weeks [54].

#### Conclusion of ICS/LABA choice

Because there are insufficient trials of head-to-head comparison design, adequate duration (>12 weeks) or with clinically relevant outcomes, it is currently difficult to recommend any of ICS/LABA combination over another. As the body of evidence is largest for the FP/S combination, physicians are likely to continue to favour this combination. There are indications in the literature that BUD/F results in less adverse events, but there is still uncertainty on this issue.

### Long-acting anticholinergic combinations

#### Group D patients: ICS/LABA/LAMA or “Triple therapy”

Adding a LAMA (tiotriopium, TIO; glycopyrronium, GLY; or aclidinium) to ICS/LABA is called “triple therapy”. GOLD 2013 recommends for group D patients that ICS be combined with LABA or LAMA, “with some evidence for triple therapy”. Indeed, a 2011 Cochrane review [55] only found three studies, and had low confidence in drawing any conclusions for the outcomes as a result of heterogeneity and wide confidence intervals, except for an improvement of quality of life scores and lung function for triple therapy versus TIO alone. Only one small study also examined our clinically relevant question of triple therapy versus ICS/LABA, which was insufficient to draw firm conclusions.

Since the review, there have been a few new trials. Hanania et al. [56] was a 24-week, double-blinded RCT (n = 342) of TIO (18 μg OD) versus triple therapy (FP/S 250/50 μg DISKUS BD and TIO). Triple therapy improved lung function and reduced rescue inhaler use. Unlike some of its predecessors, there was no difference in exacerbations either. The adverse event profile was also similar. A similar trial of the same length and doses (n = 479) also found triple therapy to be superior in lung function, and in quality of life. This study, however, was open-label; the lack of blinding reduces our confidence in its results [57]. A smaller, 8-week RCT found no difference in exercise tolerance between triple therapy (FP/S/TIO) compared to TIO alone [58], but this study is less clinically relevant as it is too short to comment on outcomes such as exacerbations.

Two future studies have been identified, both using GLY instead of TIO, and comparing to triple therapy involving BDP/F. [59, 60] They plan to publish in 2016. The Singh study will investigate our clinically relevant question of BDP/F/GLY vs. BDP/F.

Loke and Singh [61] describe how concerns over cardiovascular events and mortality have existed around TIO for a while now, especially around the Respimat inhaler. They have not entirely been alleviated by the UPLIFT trial, since there has been new, observational data raising more concerns regarding urinary retention. Compared with the TIO HandiHaler, “some experts have argued that there is no compelling reason to choose the Respimat formulation” [61]. This may be why attention is turning to GLY instead. Meanwhile, a 3–year, multinational trial is seeking to answer the questions around Respimat once and for all [62].

#### LABA/LAMA versus ICS/LABA

We have mainly examined comparisons of the first choice column of the GOLD recommendations. Other comparisons (e.g. involving ICS/LAMA) have been less addressed in the literature, but may become commercially relevant to investigate once a company devises a new fixed combination. This could alter the GOLD first choice recommendations in the future. One such new fixed combination is a LABA/LAMA: indacaterol with glycopyrronium, also known as “QVA149”. Indacaterol is a new once-daily LABA that has recently been reviewed by Ribeiro and Chapman [63].

The ILLUMINATE trial investigated QVA149 110/50 μg OD versus FP/S 50/500 μg BD over 26 weeks (n = 253). They found significant improvements in lung function and some symptom outcomes. The lack of pneumonia events (vs. four in FP/S) and less adverse events, including exacerbations and withdrawal, seem the most promising results for future investigation [64]. This was a trial of stage 2 and 3 COPD patients with less than two exacerbations in the previous year.

Other new LABA/LAMA fixed combinations in development or awaiting regulatory approval are: vilanterol/umeclidinium, olodaterol/tiotropium, and BD formoterol/aclidinium [65].

### Inhalers

We have not yet addressed any questions regarding which inhalers to choose. A new study challenged the previous evidence that a fixed combination inhaler is better for compliance than single inhalers, finding no difference [66]. The authors caution that this may have only been because their trial population is rigorously trained, and may not reflect real-life. It does raise the possibility of not being restricted to only the fixed combination inhalers we have examined in this paper.

Clinicians may also wonder whether the type of inhaler makes any difference. In most European countries, the following inhalers are available for ICS/LABA: pMDIs and various DPIs (Aerolizer, Turbuhaler, Diskus). When looking at separate inhalers, for LABA or ICS, there's also the Novolizer DPI and nebulisers, and for ICS only, there is the additional Autohaler (breath-actuated pMDI) [67, 68]. Nebulisers are reserved for emergencies, or patients who cannot handle a pocket-sized device [67]. There has been a flurry of new DPI inhalers because of patent mortality [69], and because it is felt the pMDIs have many limitations [70]. One such new DPI device is the Rotahaler, which is currently being trialled [71]. There have been RCTs trying to establish the efficacy of MDIs over DPIs because of their relative advantages, however, including an FP/S MDI [72]. The relative advantages and disadvantages of pMDIs compared to DPIs are summarised in Table 2.

**Table 2:** Advantages and disadvantages of MDIs and DPIs

**MDI**	**DPI**
Good hand-breath coordination required (except for breath-actuated Autohaler) [68]	No need for coordination of inhalation and actuation [70]
Addition of spacer does not completely fix this	Does not require a spacer [70]
May be unsuitable for elderly, confused, or patients with hand rheumatologic conditions	Easier to use than MDI [67, 70]
Suitable for tracheostomy, intubation [72]	Breath-actuation difficult in patients with low inspiratory effort (hyperinflation, muscular weakness) [67]
Concerns about HFA-propellant environmental friendliness [72]	Lactose carrier: contra-indicated in lactose allergy [68, 72]
Drugs may settle (shake before use) [68]	

RCT comparing inhaler devices are scarce and systematic reviews have found no difference in clinical efficacy [67]. These are, however, limited in generalisability to real-life situations, as only patients able to correctly use these inhalers, are selected for RCTs [67]. Decision-making should, therefore, involve a range of different factors, including: the individual patient; price and availability; similarity to other inhalers the patient uses; ability for the health carer to correctly train the patient; ability of the patient to handle the inhaler; patient preference [67].

## Exacerbations of COPD: systemic steroids

For COPD acute exacerbations, the GOLD treatment remains respiratory support, SABA with or without SAMA, systemic steroids, and antibiotics if a bacterial infection is suspected. Prednisolone 30 – 40 mg per day for 10 – 14 days is suggested by GOLD; [1•] however, evidence has changed over the last year to favouring a 5-day course. The REDUCE trial (n = 296) showed that 5 days of 40 mg of prednisolone was non-inferior to 14 days, with no difference in time to first exacerbation within 6 months, mortality, recovery of lung function, or treatment-related adverse events [73•]. This trial will help standardise steroid regimens, and reduce cumulative exposure and risk of toxicity for frequent exacerbators [74]. Another trial last year investigated whether intravenous (IV) treatment was better than methylprednisolone taken orally, but found the reverse [75], following on from a trial showing superiority of IV methylprednisolone over IV hydrocortisone in lung function only, but not recurrence of exacerbations [76].

The REDUCE trial highlighted the poor prognosis of COPD exacerbation, even when treated with steroids, and the need to find new management options [74]. One such option was etanercept (a tumour necrosis factor α [TNFα] antagonist), but a small clinical trial, limited by its short time-to-follow-up, found it was not more effective than prednisone in the treatment of exacerbations [77].

Meanwhile, GOLD suggests nebulised budesonide as an alternative [1•]. Indeed a non-blinded trial of 86 hospitalised patients compared nebulised budesonide of 2 mg and of 4 mg (BD) with IV methylprednisolone 40 mg up until discharge and found no difference between the groups in spirometry or arterial blood gas samples [78], but this finding requires further study before clinical use.

In an intensive care (ICU) setting, last year, a pairwise, retrospective, case-control matching study (n = 32) of exacerbating patients requiring mechanical ventilation or ICU found that steroids taken orally reduced the time of mechanical ventilation [79], whereas for severe exacerbations, a randomised evaluation showed that prednisone did not improve ICU mortality or patient-centred outcomes. It significantly increased the risk of hyperglycaemia [80]. Lastly, a new large cohort study (n = 17,239) suggested that there may be benefits to using low doses of methylprednisolone (<240 mg/day) over higher regimens, but randomised trials are needed to confirm this [81].

## Corticosteroid responsiveness

There is still a concern about a significant group of COPD patients that are less steroid-responsive. Papers last year suggested that steroid resistance could be reversed using theophylline, phosphoinositide 3-kinase δ inhibitors, or LABAs [82], using adjunctive adenosine A2B-receptor agonist therapy [83], using cyclosporin or CRAC channel inhibitor Synta 66 [84], the novel macrolide solithromycin [85], a p38 mitogen inhibitor [86], or PDE4 inhibitors [87, 88]. However, these results remain on the research agenda and have no clinical implications yet.

## Steroid withdrawal

With questions surrounding the effectiveness of steroids in COPD, withdrawal trials have been performed in the past to evaluate whether patients treated with ICS, fared better without them. A 2011 systematic review of such trials found no evidence that withdrawing patients resulted in important deterioration in outcomes in practice [89]. A protocol was published this year for a big study addressing this issue. The WISDOM study (n = 2,456) will investigate stepwise steroid withdrawal versus continuation of treatment in GOLD stage 3 and 4 patients who are exacerbators, with as primary outcome the first exacerbation up to 52 weeks [90].

## Heterogeneity of COPD

The GOLD guidelines moved away in 2011 from purely lung-function measurements as a method of predicting response to treatment, to a more phenotypic classification of COPD groups, by introducing exacerbation risk and mMRC or CAT scores (and later CCQ). Recently, researchers have started going back to exploring splitting COPD into several different phenotypes [91, 92]. Not all of these phenotypes are clinically useful at the moment, and there is a lack of big studies validating them or testing different treatment regimes. Exceptions have included Rennard [93] and Albert. [94] Several countries’ COPD guidelines have already tried to account for some phenotypes where we have evidence that a different pharmacological management is beneficial, including Canada’s 2007 guidelines [95], Japan [96], and Spain [97]. Miravitlles et al. [92] suggest four clinically relevant phenotypes: infrequent exacerbators, frequent exacerbators with either emphysema or chronic bronchitis separately, and a COPD-asthma overlap type. Of these, except for the first group, all are amenable to ICS. For exacerbations, based on Bafadhel et al. [98], they suggest distinguishing non-eosinophilic exacerbators from eosinophilic, which improve on systemic steroid, as opposed to the former group which may worsen on steroids compared to placebo. Bafadhel et al. [98] is a 2-week, double-blind interventional trial, where there was a significantly greater improvement in the chronic respiratory questionnaire (CRQ) of patients with exacerbations that were eosinophilic-negative and treated with placebo than if treated with prednisolone. There was also a significant increase in treatment failures on prednisolone than placebo. Also, there was no significant difference in CRQ of patients that were treated based on eosinophils compared to the patients that were treated without eosinophilic guidance, while it reduced the likelihood of being treated with prednisolone by half. However, the authors themselves recognise that larger studies are needed [98]. This evidence would suggest that this classification is not ready for use in clinical practice yet.

It is likely that, in future, the GOLD guidelines will adjust even more to account for different phenotypes. Meanwhile, more research into associated genes has identified further groups [91], bringing individualised therapy based on genetic phenotyping closer to reality [99].

## Conclusion

New GOLD guidelines take into account exacerbation risk and quality of life scores. It is possible that, in future, COPD phenotypes will be sub-divided further. ICS are only recommended in combination with a LABA and/or LAMA. The type of ICS chosen should be carefully considered based on the patient’s need for a reduction in exacerbations, balanced against the risk of pneumonia. More head-to-head trials are needed. However, such trials are unlikely due to both the sample size and length that would be needed to show a difference in effect, and the lack of commercial incentive for investing in such trials. The type of inhaler should be chosen based on the individual patient’s needs and preferences. For exacerbations of COPD, systemic steroids should now only be given for 5 days instead of 10 – 14 days, but more research is needed into alternative therapies.
